# Collateral status evaluation coupled with time window by dynamic axial computed tomographic angiography with a focus on the middle cerebral artery for mechanical thrombectomy

**DOI:** 10.1186/s12883-021-02284-8

**Published:** 2021-06-22

**Authors:** Takahisa Mori, Kazuhiro Yoshioka, Wataru Mori, Yuhei Tanno

**Affiliations:** Department of Stroke Treatment, Shonan Kamakura General Hospital, Okamoto 1370-1, Kamakura City, Kanagawa 247-8533 Japan

**Keywords:** Ischemia, Stroke, Computed tomography angiography, Thrombectomy, Reperfusion

## Abstract

**Background:**

Dynamic axial computed tomographic angiography (dynax–CTA), covering a thin width, with a focus on the bilateral middle cerebral artery (MCA), can quickly visualize the internal carotid artery (ICA) or MCA occlusion. We aimed to investigate whether dynax–CTA appropriately evaluated the collateral status coupled with the upper limit of the onset-to-reperfusion (OtR) time to achieve a major neurological improvement (MNI) at a 24-h follow-up examination after mechanical thrombectomy (MT).

**Methods:**

We included acute ischemic stroke patients admitted from 2018 to 2020 who underwent dynax–CTA on admission and emergent MT for ICA or MCA occlusion. We performed dynax–CTA using an 80-row CT scanner and acquired 25 volume scans, consisting of 40 images of 1-mm thickness and 4-cm width. We classified the collateral status as good, intermediate, and poor based on MCA branch opacification. We evaluated the collateral status and the upper OtR time limit to achieve MNI.

**Results:**

Forty-eight patients met our inclusion criteria. Dynax–CTA findings demonstrated MCA and ICA occlusion in 30 and 18 patients, respectively. The collateral status was good, intermediate, and poor in four, 25, and 19 patients, respectively. The upper limits of the OtR time for MNI were 3.63, 8.08, and 8.67 h in patients with poor, intermediate, and intermediate or good collateral status, respectively.

**Conclusions:**

Dynax–CTA appropriately evaluated the collateral status coupled with the upper limit of the OtR time before performing MT.

**Supplementary Information:**

The online version contains supplementary material available at 10.1186/s12883-021-02284-8.

## Background

Whole head dynamic computed tomographic angiography (CTA), referred to as four-dimensional CTA (4D-CTA), can visualize vascular flow dynamics in bilateral cranial vessels; it is a promising method for diagnosis and patient selection before performing mechanical thrombectomy (MT) in cases of acute ischemic stroke (AIS) [1]. However, the long processing time of dynamic CTA, because of its ability to produce large volumetric data, limits its applicability before MT [2]. Perfusion CT is used in many stroke centers to identify candidates for MT [3, 4]. However, it requires vendor-specific algorithms to generate images [5], which are not standardized and can vary across research facilities. Dynamic CTA, but not necessarily that encompassing the whole head, is available in most facilities, and can be standardized. Dynamic axial CTA (dynax–CTA), instead of covering the whole head, covers a 4-cm width with a focus on the middle cerebral artery (MCA) axis, reduces the volumetric data generated, and shortens the processing time of dynamic CTA. Moreover, infero-superior view of dynax–CTA images can visualize the internal carotid artery (ICA) or MCA occlusion for patient selection before MT [6]. It is critically important to reduce the onset-to-reperfusion (OtR) time to achieve better functional outcomes even in patients with a small baseline ischemic core size [7–9]. In contrast, the time window for MT is extended to 24 h in patients with a mismatch between clinical deficit and an infarct volume on perfusion CT or diffusion-weighted magnetic resonance imaging (MRI). However, the infarct volume was measured with the use of vendor-specific automated software [4], and it remains difficult to know the upper limit of the OtR time based on perfusion CT images on admission. In cases where dynax–CTA with a short processing time and without a vendor-specific software can provide an appropriate collateral flow status coupled with the OtR time for MT, it could be a useful method in many facilities.

In the present study, we investigated whether dynax–CTA appropriately evaluated the collateral status coupled with the upper limit of the OtR time before performing MT.

## Methods

For this retrospective cross-sectional study, we included AIS patients who met the following criteria: 1) admitted to our institution within 24 h after stroke onset from August 2018 to March 2020; 2) underwent dynax–CTA covering a 4-cm width with a 1-mm thickness on admission; and 3) underwent MT for ICA or MCA occlusion within 24 h after stroke onset. We excluded patients with AIS who underwent MRI before dynax–CTA or MT without undergoing dynax–CTA.

### Evaluation

We evaluated the following patients’ characteristics: onset-to-door (OtD), door-to-imaging (DtI), imaging-to-puncture (ItP), puncture-to-reperfusion (PtR), and OtR times [10]; the Alberta Stroke Program Early CT Score (ASPECTS); the collateral status based on dynax–CTA; successful reperfusion of modified thrombolysis in cerebral infarction grade 2b or 3 [10]; the NIH Stoke Scale (NIHSS) score on admission and at 24 h after MT performance; and major neurological improvement (MNI) at the 24-h follow-up defined as a 50% decrease in the NIHSS score at 24 h after MT [11]. Furthermore, we evaluated the association between the MNI and OtD, DtI, ItP, PtR, and OtR times. In addition, we estimated the upper limits of the significant time to achieve MNI by using the area under the receiver operating characteristic curve, which was derived from the receiver operating characteristic (ROC) curves of the logistic regression model [11], and the interrater reliability for the collateral status on dynax–CTA.

### Dynax–CTA

**Fig. 1:**
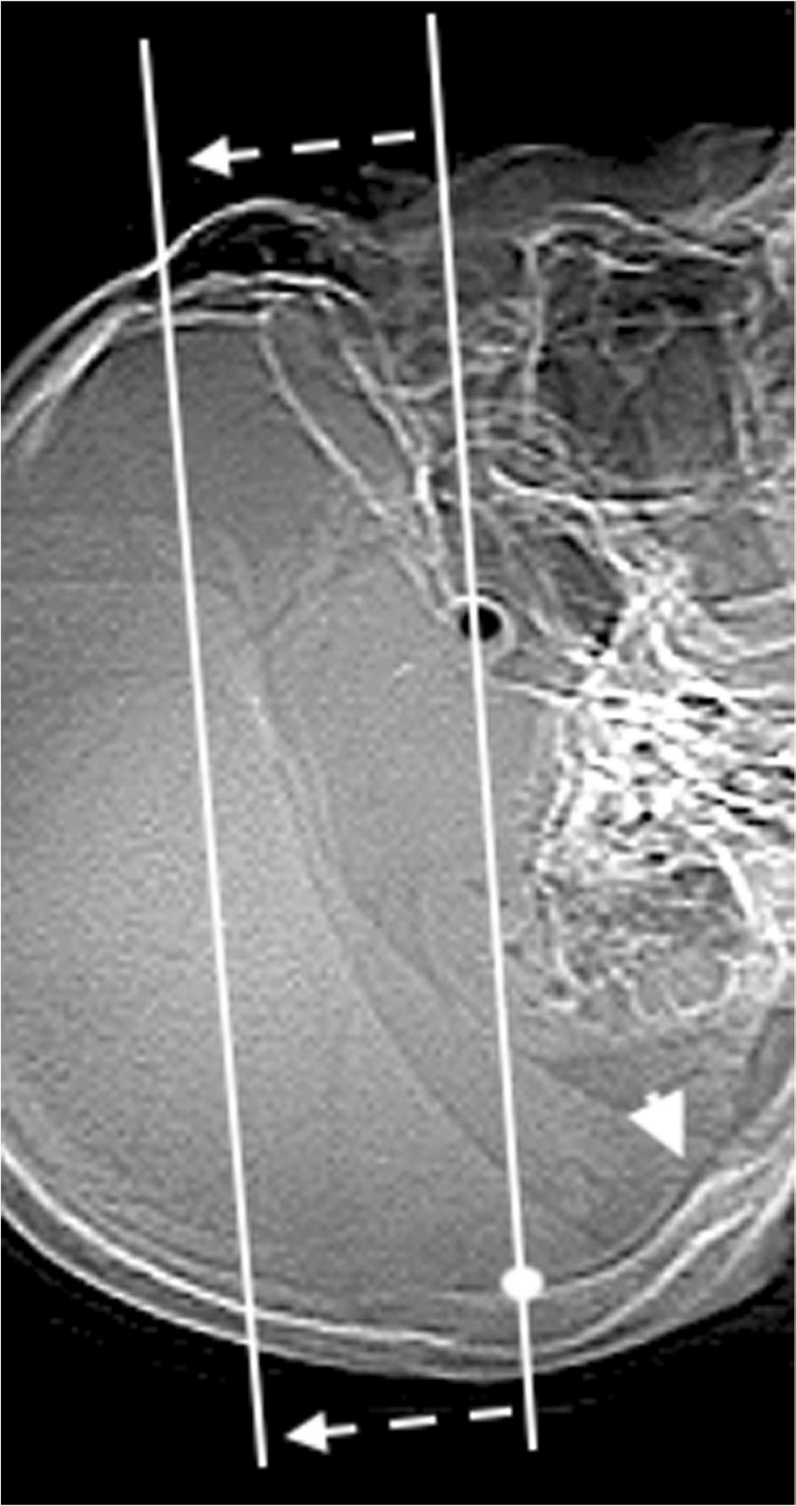
A CT scout image. The CT acquisition started from the line (inferior white line) connecting the pituitary fossa (black dot) with the point (white dot) 2 cm upward from the internal occipital protuberance (arrowhead) by tilting the gantry. CT scanning was performed with 4-cm coverage (dashed arrows and superior white line). CT, computed tomography


We performed image acquisition using the 80-row area detector CT scanner (Aquilion PRIME, Canon Medical Systems, Otawara, Tochigi, Japan) [6]. The acquisition began from the line connecting the pituitary fossa with the point located at 2 cm upward from the internal occipital protuberance by tilting the gantry to focus on the MCA axis (Fig. 1). Volume scanning of bilateral MCAs was performed by injecting 40 mL of a non-ionic contrast medium (iopamidol; 370 mg/mL) at a rate of 4 mL/s. CTA by 1-s single rotation and 1-s intermittent dataset scans were acquired at 80 kVp. After injection of the contrast medium, 25 intermittent volume scans at 100 mA were acquired every other second at 8 s or later during the possible arrival of the contrast medium into the intracranial arteries. Each volume scan consisted of 40 images of 1-mm thickness with a z-axis coverage of 4 cm, as 80-row detectors of 0.5-mm thickness generate 80 images with 0.5-mm thickness scanning, indicating the production of double volumetric data. The total computed tomography dose index volume (CDDIvol) was 172.5 mGy, and the total dose length produced (DLP) was 690 mGy ∗ cm/25 scans and 4 cm. The volumetric data of 1000 images were automatically transferred without subtraction to a workstation (Ziostation2, Ziosoft, Inc., Tokyo, Japan). After the dynax–CTA acquisition, we performed helical scanning for neck and head CTA. Helical CT scanning was performed by injecting 38 mL of a non-ionic contrast medium at a rate of 3.8 mL/s. The total CTDIvol was 253 mGy and the total DLP was 380 mGy ∗ cm. Approximately 340 images were automatically transferred to the workstation. We generated dynax–CTA images of the bilateral MCAs (Figures S1, S2, and S3) and maximum intensity projection for neck and head CTA (Figure S4).

**Additional file 1: Fig. S1.** Dynax–CTA movie demonstrating good collateral status in a patient with right MCA M2 occlusion. CTA, computed tomography angiography; MCA, middle cerebral artery.


**Additional file 2: Fig. S2.** Dynax–CTA movie demonstrating intermediate collateral status in a patient with right intracranial ICA occlusion and right MCA M1 occlusion. CTA, computed tomography angiography; ICA, internal carotid artery; MCA, middle cerebral artery.


**Additional file 3: Fig. S3.** Dynax–CTA movie demonstrating poor collateral status in a patient with right MCA M1 occlusion. CTA, computed tomography angiography; MCA, middle cerebral artery.

### Collateral status

**Fig. 2:**
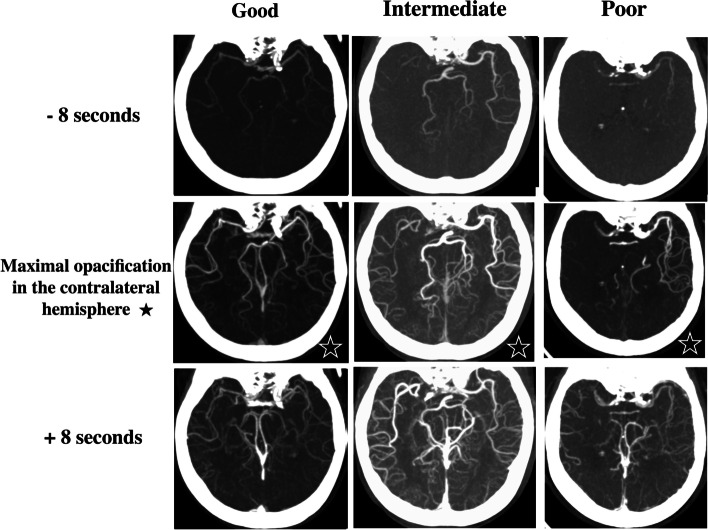
Collateral status based on dynax–CTA. Column: The collateral status was good, intermediate, and poor in the right M2 (left), right M1 (middle), and right M1 occlusion (right), respectively. Row: Time-resolved images showing 8 s (four frames) before maximal opacification of the MCA in the contralateral hemisphere (upper), maximal opacification of the MCA in the contralateral hemisphere (asterisk) (middle), and 8 s (four frames) after maximal opacification of the MCA in the contralateral hemisphere (lower). dynax–CTA, dynamic axial computed tomography angiography; MCA, middle cerebral angiography

We viewed dynax–CTA images inferosuperiorly on the workstation and classified the collateral status of the patients as good, intermediate, or poor collateral based on the MCA branches’ opacification by dynax–CTA (Fig. 2, Figures S1, S2, and S3) (Table 1). A good, intermediate, and poor collateral status, as evaluated by dynax–CTA, was equivalent to grade 4, 2 or 3, and 0 or 1, respectively, according to the American Society of Interventional and Therapeutic Neuroradiology/Society of Interventional Radiology (ASITN/SIR) scale on angiography [12].

**Table 1:** Collateral status according to vascular flow dynamics on dynax–CTA

**Good**	Compared to visualization of the M2 or M3 branches in the asymptomatic contralateral hemisphere, the M2 or M3 branches were visualized almost without delay within the ischemic territory
	in the symptomatic hemisphere, and prominence and extent was about 70% or more of those in the contralateral hemisphere
**Intermediate**	Compared to visualization of the M2 or M3 branches in the asymptomatic contralateral hemisphere, the M2 or M3 branches were visualized with a short delay within the ischemic territory
	in the symptomatic hemisphere, and prominence and extent was about 30 to 70% of those in the contralateral hemisphere
**Poor**	Compared to visualization of the M2 or M3 branches in the asymptomatic contralateral hemisphere, the M2 or M3 branches were visualized with a long delay within the ischemic territory
	in the symptomatic hemisphere, and prominence and extent was about less than 30% of those in the contralateral hemisphere

*dynax–CTA* Dynamic axial computed tomography angiography

### Mechanical thrombectomy

We made a clinical decision for MT in patients with an NIHSS score ≥ 6, ICA or MCA occlusion seen on neck and head CTA and dynax–CTA, ASPECTS score ≥ 6, no intracranial hemorrhage on pre-contrast CT, and within 24 h of OtD. MT was determined when patients with poor, intermediate, and good collateral presented an OtD time of 8, 16, and 24 h, respectively. If the OtD time was < 3.5 h, intravenous alteplase (0.6 mg/kg) was administered [13]. We obtained written informed consent from the patient or the patient’s legally authorized representative before performing MT. We performed MT under local anesthesia, introduced a balloon-guide catheter, and used an aspiration catheter, a stent-retriever, or a combination of both [14, 15]. We defined successful recanalization as a modified treatment with a cerebral infarction score of 2b or 3 [10].

### Interrater reliability

Two raters (KY, WM) independently assessed dynax–CTA images in randomly chosen participants using the three collateral status system (i.e., good, intermediate, and poor collateral status).

### Statistical analysis

Non-normally distributed continuous variables are expressed as medians and interquartile ranges (IQRs). Differences between the unpaired variables were compared using the chi-square or Fisher’s exact test for categorical variables and the Wilcoxon rank-sum test for non-parametric data. Differences between the three collateral status groups were compared using the Wilcoxon rank-sum test; this test was also used to compare the paired variables for non-parametric data. Interrater reliability was measured using Cohen’s kappa coefficient (k). The Youden’s index was used to determine the optimal cut-off values on multiple logistic analysis. A *p*-value < 0.05 was considered statistically significant. We used the JMP software (version 15.2; SAS, Cary, NC, USA) to make the statistical analysis.

## Results

During the study period, 524 AIS patients were admitted within 24 h after stroke onset. Among them, dynax–CTA findings revealed ICA or MCA occlusion in 91 patients, and 53 of them underwent MT within 24 h after stroke onset. Five of the 53 patients were excluded from analysis because they underwent MRI before dynax–CTA. Finally, 48 patients met our study inclusion criteria. Among them, 15 patients underwent intravenous alteplase therapy. Table 2 summarizes the patients’ characteristics. Dynax–CTA demonstrated MCA and ICA occlusion in 30 and 18 patients, respectively. Among the 18 patients with ICA occlusion, two underwent carotid stenting for proximal carotid artery stenosis followed by MT for distal ICA occlusion. Successful recanalization was achieved in 46 patients (95.8%). The NIHSS score was 19 (15–22) on admission and 4 (1–12.5) at 24 h after MT (*p* < 0.0001). Thirty-four (70.8%) of the 48 patients achieved MNI.

**Table 2:** Comparison between three groups of collareral status

dynax-CTA: collateral status	All	Poor	Intermediate	Good	*P*
n	48	19	25	4	
Age, median (IQR) years	81.5 (77–86)	83 (76–87)	81 (79–83)	73 (66–83)	ns
Female (sex)	34 (70.8%)	10 (52.6%)	20 (80%)	4 (100%)	ns
ASPECTS, median (IQR)	10 (7–10)	10 (6–10)	10 (7.5–10)	9.5 (7.5–10)	ns
OtD, median (IQR) hours	2.62 (0.84–8.32)	1.67 (0.75–4.98)	3.65 (0.94–10)	5.73 (2.44–11.13)	ns
DtI, median (IQR) hours	0.38 (0.32–0.5)	0.37 (0.32–0.45)	0.38 (0.28–0.53)	0.38 (0.32–1.23)	ns
ItP, median (IQR) hours	0.93 (0.64–1.29)	0.93 (0.58–1.27)	0.8 (0.64–1.39)	1.21 (0.85–1.33)	ns
PtR, median (IQR) hours	0.66 (0.45–1.1)	0.7 (0.47–1.2)	0.59 (0.46–1.04)	0.65 (0.27–1.93)	ns
OtR, median (IQR) hours	5.38 (3.24–10.02)	4.3 (2.85–8.58)	6.05 (3.33–11.63)	8.3 (6.27–12.89)	ns
NIHSS adm, median (IQR)	19 (15–22)	19 (17–22)	18 (14.5–22)	16 (14–24.8)	ns
NIHSS at 24, median (IQR)	4 (1–12.5)	7 (2–20)	2 (0–8.5)	5 (1–11.25)	ns
Alteplase therapy	15 (31.3%)	6 (31.6%)	8 (32.0%)	1 (25.0%)	ns
Successful reperfusion (mTICI 2b/3)	46 (95.8%)	18 (94.7%)	24 (96.0%)	4 (100%)	ns
MNI, n (%)	34 (70.8%)	10 (52.6%)	21 (84.0%)	3 (75%)	ns

*dynax–CTA* Dynamic axial computed tomography angiography, *IQR* Interquartile range, *ItP* Imaging-to-puncture time, *MNI* Major neurological improvement, *mTICI* Modified thrombolysis in cerebral infarction, *NIHSS* National Institutes of Health Stroke Scale, *OtD* Onset-to-door time, *OtR* Onset-to-reperfusion time

There were no statistical differences in the time variables between the three collateral statuses; however, the median OtD time was the shortest in the poor collateral status group (Table 2). To achieve MNI, the ASPECTS of a perfect 10 and a short PtR time were critical in those with poor collateral status, and a short OtR time was critical in those with poor, intermediate, and intermediate or good collateral status (Table 3). Four patients were included in the good collateral status group, and statistical analysis to identify significant variables was not performed. Therefore, the patients in the good collateral status group were incorporated in the intermediate collateral status group for statistical analysis (Tables 3 and 4).

**Table 3:** Time variables and major neurological improvement

	NIHSS score decrease ≥ 50% at 24 h	NIHSS score decrease < 50% at 24 h	*P*
Collateral status: Poor			
n	10	9	
ASPECTS	10 (10–10)	6 (3.5–8)	< 0.001
OtD hours	1.07 (0.63–3.74)	1.92 (1.18–8.73)	ns
DtI hours	0.35 (0.26–0.41)	0.43 (0.33–0.51)	ns
ItP hours	0.83 (0.55–0.99)	1.21 (0.78–2.0)	ns
PtR hours	0.52 (0.38–0.72)	1.15 (0.74–1.68)	< 0.01
OtR hours	3.26 (2.50–5.35)	5.48 (4.14–11.25)	< 0.05
Collateral status: Intermediate			
n	21	4	
ASPECTS	10 (7–10)	9 (7.5–9.8)	ns
OtD hours	1.83 (0.86–7.96)	11.58 (9.15–13.52)	< 0.05
DtI hours	0.38 (0.28–0.54)	0.44 (0.38–0.52)	ns
ItP hours	0.8 (0.61–1.39)	1.01 (0.72–2.08)	ns
PtR hours	0.55 (0.46–0.95)	0.96 (0.5–1.36)	ns
OtR hours	5.12 (2.9–9.53)	13.98 (11.02–17.20)	< 0.05
Collateral status: Good			
n	3	1	
ASPECTS	10 (7–10)	9	
OtD hours	7.33 (4.12–12.4)	1.88	
DtI hours	0.42 (0.33–1.5)	0.32	
ItP hours	1.15 (0.75–1.35)	1.26	
PtR hours	0.33 (0.25–0.97)	2.25	
OtR hours	8.67 (7.93–14.3)	5.72	
Collateral status: Good or Intermediate			
n	24	5	
ASPECTS	10 (7.3–10)	9 (8–9.5)	ns
OtD hours	3.49 (0.91–9.02)	11.08 (5.19–13.04)	< 0.05
DtI hours	0.39 (0.28–0.55)	0.38 (0.35–0.51)	ns
ItP hours	0.81 (0.63–1.38)	1.24 (0.74–1.81)	ns
PtR hours	0.54 (0.43–0.96)	1.26 (0.55–1.82)	ns
OtR hours	5.64 (3.26–10.40)	13.97 (7.88–16.13)	< 0.05

All values are represented as median (interquartile range) unless specified otherwise

*OtD* Onset-to-door time, *ItP* Imaging-to-puncture time, *OtR* Onset-to-reperfusion time

**Table 4:** A 50% or more decrease in NIHSS from baseline to 24 h using receiver operating curves by logistic regression analysis

	N	Sens (%)	Spec (%)	PPV (%)	OR	*P*	AUC	AICc	BIC
Collateral status: Poor									
OtR (≤ 3.63 vs > 3.63) h	19	70	88.9	87.5	0.73 (0.44–0.98)	0.11	0.79	26.2	27.4
Collateral status: Intermediate									
OtR (≤ 8.08 vs > 8.08) h	25	76.2	100	100	0.58 (0.24–0.85)	0.05	0.92	16.2	18
Collateral status: Intermediate or Good									
OtR (≤ 8.67 vs > 8.67) h	29	75	80	94.7	0.7 (0.52–0.94)	0.03	0.82	24.7	26.9

*AUC* Area under the curve, *AICc* Corrected Akaike information criterion, *BIC* Baysian information criterion, *OR* Odds ratio, *OtR* Onset-to-reperfusion time, *NIHSS* National Institutes of Health Stroke Scale, *PPV* Positive predictive value, *Sens* Sensitivity, *Spec* Specificity

ROC curves, constructed by logistic regression analysis for MNI, showed that the upper limits of the OtR time were 3.63, 8.08, and 8.67 h in patients with poor, intermediate, and intermediate or good collateral status (Table 4), respectively. The therapeutic time window was the narrowest in those with poor collateral status and more extensive in those with intermediate or good status. In 19 patients with poor collateral status, seven out of seven patients with an ASPECTS of 10 and OtR time of ≤ 3.63 h achieved MNI (positive predictive value, 100% [7/7]; negative predictive value, 75% [9/12]). In 25 patients with intermediate collateral status, 16 out of 16 patients with an OtR time of ≤ 8.08 h achieved MNI (positive predictive value, 100% [16/16]; negative predictive value, 44.4% [4/9]). The collateral status was coupled with the OtR time to achieve MNI. Its evaluation was based on dynax–CTA findings, which provided information regarding the therapeutic time window for MNI after undergoing successful MT.

### Interrater reliability

We found that the interrater reliability was substantial for collateral status after performing dynax–CTA (*n* = 22, k = 0.773, *p* < 0.0001).

## Discussion

Dynax–CTA is a time-resolved CTA method, which generates 25 sequential images over 50 s with our protocol. Dynax–CTA visualized the flow dynamics of the MCA in the bilateral hemispheres. Performing dynax–CTA with substantial interrater reliability, covering a 4-cm width, could appropriately provide the collateral status coupled with the upper limit of the OtR time for MNI.

Proper head position for dynax–CTA with a thin width was required to obtain the appropriate dynamic images of the MCAs [16]. Unless head position is proper, useful information cannot be scanned.

Time-density curves of dynamic CT on admission were used to estimate the collateral flow status and clinical outcome in AIS patients [17]. A poor collateral flow status was a predictor of unfavorable clinical outcomes [17]. Time-intensity curves of dynamic MRI were used to estimate the collateral flow status and clinical outcome in AIS patients with ICA or MCA occlusion [18]. The collateral score on dynamic CTA was reported to estimate the final infarct volume [1]. Indeed, time-intensity curves or the collateral scores comparing the affected territory with the contralateral territory are objective indices, but flow dynamics of dynax–CTA may allow the recognition of the differences in flow velocity and vascular volume between them.

We performed 25 volume scans in this study, and an arbitrary number of scans can be determined in different facilities. Multiphase CTA is an excellent tool to identify candidates for MT [11, 19]. However, triphasic CTA may fail to identify candidates because each of the three phases is 8 s apart, corresponding to three images in 16 s [11]. In our protocol, nine images were acquired in 16 s, and they may allow the recognition of flow differences between the bilateral MCA territories. In total, 80 mL of contrast material was injected in triphasic CTA, and 78 mL of contrast material for dynax–CTA and neck and head CTA was used in our protocol.

Volume scanning performed for dynax–CTA can provide perfusion CT images on the workstation. Perfusion CT has been used to delineate the infarct core and penumbra in several clinical trials [3–5, 20]. However, the use of dynax–CTA has not been reported in randomized clinical trials. A strategy combining CTA and perfusion CT was more cost-effective than that involving CTA alone during the patient selection of alteplase treatment [21]; however, the cost-effectiveness of dynax–CTA has not been established. Image quality is occasionally affected by patient motion in perfusion CT but not always in dynamic CTA. In addition, perfusion CT requires a vendor-specific application to decide whether to perform MT [4], whereas dynax–CTA does not; dynax–CTA images can be recognized easily and assist an intuitive decision of MT. Furthermore, core and penumbra estimation using automated software packages shows significant variation and should therefore be used with caution [22]. When the 64-row, 80-row, and 128-row CT scanners, and those with more rows can perform volumetric scanning, dynax–CTA can be performed without a vendor-specific application. Therefore, dynax–CTA covering a thin width with a 1-cm thickness could be a first-line tool to evaluate the collateral status in many facilities.

Our study had several limitations. A small number of patients were included, and the study had retrospective and cross-sectional design. Moreover, it was performed in a single center. A multidetector CT, different from the 80-row CT scanner, might be more useful in many facilities, and there might be a more appropriate definition of the collateral status on dynax–CTA. The presence of tandem lesions and further development of endovascular devices for MT may also affect MNI. A prospective study performed in patients from multiple stroke centers is required to confirm the effectiveness and feasibility of dynax–CTA.

## Conclusions

In conclusion, dynax–CTA, covering a thin width, could appropriately provide collateral status evaluation coupled with the upper limit of the OtR time before performing MT.

## Supplementary Information

**Additional file 4: Fig. S4.** Neck and head CTA in the same case as the intermediate case in Figure S2. CTA, computed tomography angiography.

## Data Availability

The datasets generated during and/or analysed during the current study are available from the corresponding author on reasonable request.

## Abbreviations

4D-CTA: Four-dimensional computed tomographic angiography
AIS: Acute ischemic stroke
DtI: Door-to-imaging time
dynax–CTA: Dynamic axial CTA
ICA: Internal carotid artery
ItP: Imaging-to-puncture time
OtD: Onset-to-door time
OtR: Onset-to-reperfusion time
MCA: Middle cerebral artery
MNI: Major neurological improvement
NPV: Negative predictive value
PPV: Positive predictive value
PtR: Puncture-to-reperfusion time
