# Supplementary figures and images for: Collateral status evaluation coupled with time window by dynamic axial computed tomographic angiography with a focus on the middle cerebral artery for mechanical thrombectomy
Source: BMC Neurol. 2021 Jun 22;21:230. doi: 10.1186/s12883-021-02284-8 (PMC8220685; doi:10.1186/s12883-021-02284-8)

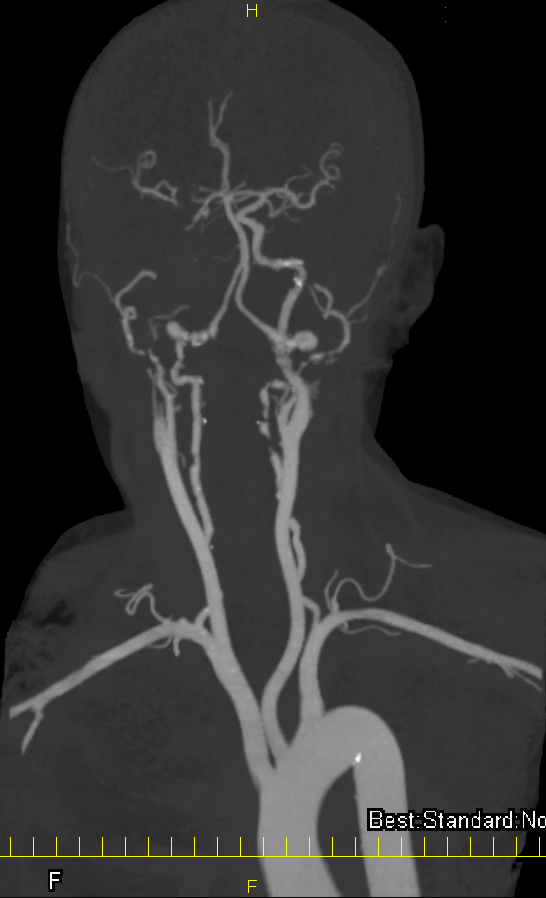

Supplement: Supplementary file 4 — Additional file 4: Fig. S4. Neck and head CTA in the same case as the intermediate case in Figure S2. CTA, computed tomography angiography. [file 12883_2021_2284_MOESM4_ESM.tif]
